# The Year of the Wisent

**DOI:** 10.1186/s12915-016-0329-3

**Published:** 2016-11-18

**Authors:** Johannes A. Lenstra, Jianquan Liu

**Affiliations:** 1 Faculty of Veterinary Medicine, Utrecht University, Yalelaan 104, 3584 CM Utrecht, The Netherlands; 2 Laboratory of Grassland Agro-Ecosystem, College of Life Science, Lanzhou University, Lanzhou, 730000 China

## Abstract

Delving into European prehistory, two recent studies analyze ancient DNA from bison species depicted by our ancestors on the walls of their caves. The DNA tells a story of migrations driven by climate change but leaves some mystery clouding the genetic descent and climate preference of the still-extant wisent, otherwise known as the European bison.

See research articles:

https://bmcbiol.biomedcentral.com/articles/10.1186/s12915-016-0317-7

http://www.nature.com/articles/ncomms13158

## Commentary

**Fig. 1:**
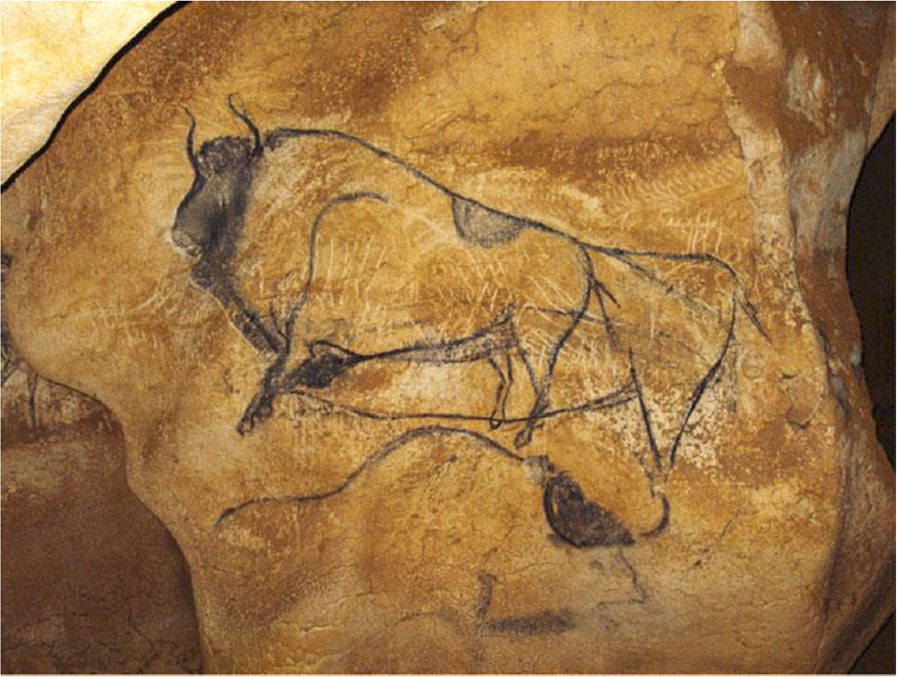
Cave paintings in Chauvet-Pont d’Arc, Ardèche, France, supposed to show a wisent (upper painting and a steppe wisent (lower painting), respectively. Both paintings are dated at around 36 kya. Printed with permission of the Centre National de Préhistoire, France. (Copyright: French Ministry of Culture and Communication, archeologie.culture.fr/chauvet; Arnaud Frich, Centre National de Préhistoire/MCC)

It is the Year of the Wisent! Already four papers have appeared on this ‘iconic’, ‘emblematic’ or ‘charismatic’ member of the bison family, which figured in an early success story of animal conservation. Rescued from extinction almost 100 years ago, the species was revived from just 12 zoo animals to generate large herds, which now roam across several European wildlife reservations. In earlier eras, as ice sheets advanced and retreated across Europe up until about 15,000 years ago, the ancestors of the wisent would have crossed paths with the now extinct Steppe bison (Fig. 1) and with the ancestors of the cattle we later domesticated. The eventful history of the wisent is now being reconstructed by several research groups, but the quest to pin down its ancestry has produced conflicting answers [1, 2].

One anomaly that any phylogeny must account for is why wisent mitochondrial DNA (mtDNA) resembles the mtDNA of taurine and zebu cattle more closely than the mtDNA from the American bison that it so obviously resembles. We reported this in 2004 [3] and proposed a hybrid origin of the wisent due to bison bulls mating successfully with aurochs cows (the ancestors of modern cattle). Since even today the American bison, the European wisent and domestic cattle are cross-fertile, it is an obvious possibility that hybridization has played a role in the evolution of these species. We favored the hybrid origin hypothesis after taking into account the social structure of herd species, with males fighting for access to females creating opportunities for bulls from a nearby related species to become dominant, especially if better adapted. But an alternative possible explanation for the aurochs-like mtDNA of the wisent is the existence of two distinct mitochondrial variants in the ancestral population that gave rise to both cattle and bison and their assortment by chance in subsequent speciation events. The hypothesis of a hybrid origin of the wisent awaited testing by a study of their genomic and/or ancient DNA (aDNA).

This wait is now over. Two recent multi-author papers [1, 2] have successfully met the challenge of capturing and sequencing DNA from ancient wisent samples in order to reconstruct their ancestry. These reports were preceded by two other studies of the origin of the wisent [3, 4], also with large molecular datasets, state-of-the-art analyses and logical interpretations. A wealth of information, but our hypothesis on the hybrid origin of the wisent has now turned into a controversy.

In the first paper published this year, the whole-genome sequence data of two wisent individuals [3] revealed that wisent and taurine cattle have mutations in common, which is evidence for hybridization of the bison and cattle lineages after the divergence of taurine cattle and zebu. This study also yielded evidence for population fluctuations during the Pleistocene associated with the successive glaciations. Next, Wecek et al. [5] looked at ancient genomes of historic samples from the extinct wild wisent. This confirmed that a small but variable part of the wisent genome originates from domestic cattle. This gene flow occurred most likely after the divergence of cattle and aurochs, but preceded the captive breeding that revived the wisent population.

The two most recent studies [1, 2], however, dig much deeper into the wisent past [4, 5]. Both describe a large aDNA dataset covering a wide geographic range, with the oldest samples dated more than 50,000 years ago. Massilani et al. [1] obtained 43 mtDNA control region sequences and 16 complete mitogenomes. Soubrier et al. [2] present sequences of 65 control region sequences and 13 complete mitogenomes as well as nuclear DNA sequences for 13 samples, accomplished by capturing 10,000 flanking sequences of SNPs from the Illumina 50K SNP panel.

The samples were identified as originating either from the wisent (*Bison bonasus*) or from the extinct Steppe wisent (*Bison priscus*). One surprising finding in both studies is the existence of two distinct wisent subpopulations, one being the ancestor of the current population and the other representing an extinct subspecies called ‘CladeX’ [2] or ‘Bb1’ [1]. Both studies also show that the population size of the wisent and steppe wisent fluctuated during the period of successive glaciations. Soubrier et al. [2] correlate this to evidence left by our artistic ancestors, who decorated the walls of their caves with paintings of several species, amongst which are wisents and steppe wisents. These paintings can be dated and by inference have recorded which species dominated during successive periods, leading Soubrier et al. to conclude that the wisent fared better than the steppe wisent in colder, tundra-like landscapes. However, this interpretation is disputed by Eva-Maria Geigl [6], the senior author of [1], who suggests the ancient wisent was better adapted to a more temperate climate and forest environment while the steppe wisent dominated in colder conditions.

**Fig. 2:**
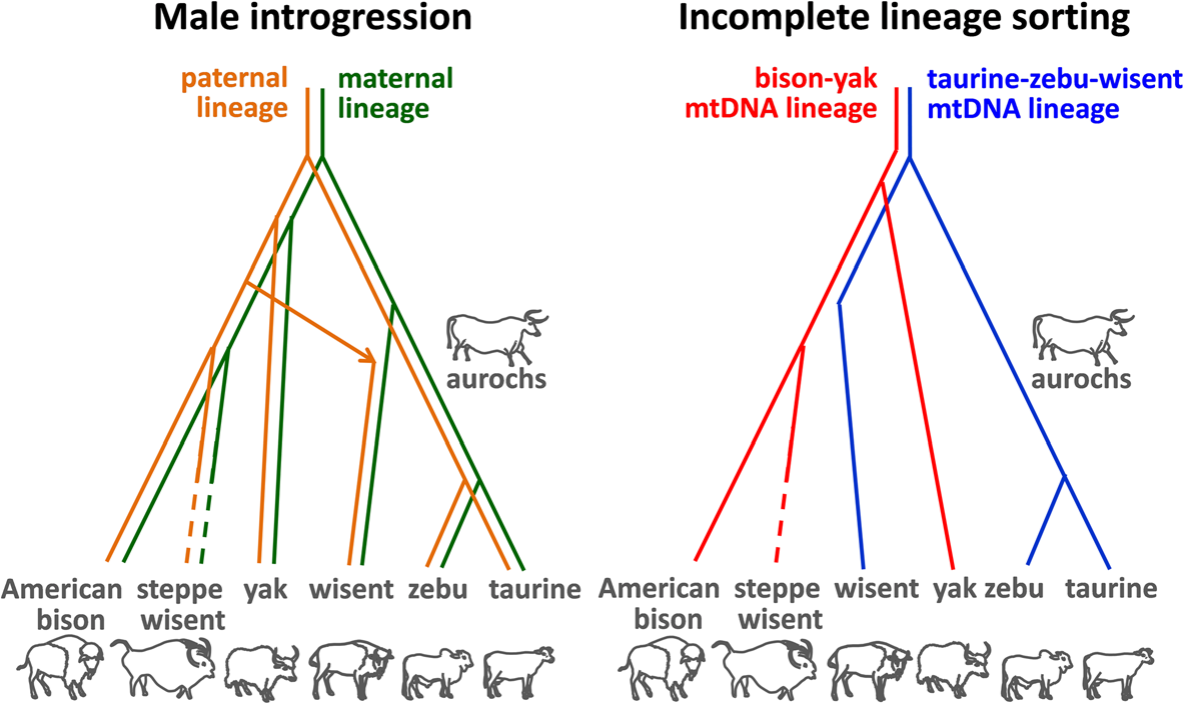
Alternative scenarios explaining the anomalous mtDNA of wisent

This is not the only disagreement between the studies [6]. The most important difference lies in the opposing explanations for the anomalous mtDNA of the wisent (Fig. 2). Soubrier et al. [2] agree with Gautier et al. [4] and our earlier hypothesis [3] about a hybrid origin of the wisent with steppe wisent and aurochs as its ancestors. Their analysis indicates a systematic ‘introgression’ of steppe wisent into aurochs herds that clearly went further than admixture by occasional genetic contacts of neighboring populations, resulting in an estimated 88% of the wisent genome contributed by the steppe wisent [2].

By contrast, on the basis of divergence times of the mtDNAs, Massilani et al. [1] propose the alternative scenario of ‘incomplete lineage sorting’ from two different mtDNA variants existing in the common bovine ancestor (Fig. 2); only one entered the aurochs lineage, while both continued for a while in the bison lineage before splitting differently between the wisent and steppe wisent/American bison branches.

Soubrier et al. [2] support their conclusions by analysis of nuclear DNA sequences. Yet the conclusions of Massilani et al. remain defensible [6]. Paleophylogenetic reconstructions are never perfect and it is easy to shoot holes in both analyses. How reliable are the geological datings or the interpolated divergence times of mtDNA [1, 6]? Why are the values of the D-statistics, a modern genomic parameter for detecting admixture, not significant for the aurochs component in wisent [1]? Predictably, authors of both studies [1, 2] were quick to spot several other potential flaws in each other’s papers [6]. However, this will not resolve the issue.

So where do we go from here? There are at least two possible quick wins. First, the groups may agree on the nomenclature for the extinct wisent subpopulation—‘CladeX’ should not last. Second, both groups may download the mtDNA sequence data of the other and see if their conclusions survive a reanalysis of a more comprehensive combined dataset. Ideally, exchange of samples and harmonization of datings may narrow down the discrepancies between the studies.

Of course, whole-genome sequencing of the ancient wisent samples will allow a more accurate analysis of admixture and divergence. For instance, Y-chromosomal variation would identify directly the parental ancestors of the wisent—the steppe wisent or maybe the American bison revisiting the continent of it ancestors [7]? Introgression is inferred from statistical parameters [2] but insignificant values would not even exclude a hybrid origin. As illustrated by the African and American zebu, which originated by crossing zebu bulls with taurine cows, systematic incrossing of exotic males during successive generations will minimize the amount of the nuclear DNA contributed by the maternal ancestor, while the mtDNA keeps its maternal origin forever [8].

Without doubt, there will be benefits from positioning the results in a wider phylogenetic context: a genomic dissection of the radiation of several bovine species including in addition to bison, wisent and steppe wisent also yak, gayal, gaur, banteng and aurochs, the latter species being the ancestor of taurine and zebu cattle. This is not only interesting for reconstructing the phylogeny of wisent, cattle and their relatives. All these species have remained cross-fertile, yet have adapted to extremely contrasting environments: from the extreme cold and hypoxia of the Himalayan plateau (yak) and the Siberian winter (taurine Yakut cattle) to the steppe, prairies and forests of the temperate zone (bison, wisent), the agriculture in the same regions (most taurine cattle) and the tropical heat (African taurine cattle, zebu, gayal, gaur, banteng).

There are evident opportunities to identify the genomic determinants of climate adaptation and the required genomic resources are now being built. Taurine and zebu cattle are well served in the successful 1000 Bull Genomes Project. A high-quality yak genome and resequencing of numerous individuals have already yielded clues on adaptation and domestication at high altitudes [9, 10]. Whole-genome sequences have been announced or are in the pipeline for bison, gayal and banteng, while, befitting the Year of the Wisent, a high-quality wisent genome sequence has been completed (Wang K, Wang L, Lenstra JA, et al., unpublished).

## References

[1] Massilani D, Guimaraes S, Brugal JP, Bennett EA, Tokarska M, Arbogast RM (2016). Past climate changes, population dynamics and the origin of Bison in Europe. BMC Biol.

[2] Soubrier J, Gower G, Chen K, Richards SM, Llamas B, Mitchell KJ (2016). Early cave art and ancient DNA record the origin of European bison. Nat Commun.

[3] Verkaar EL, Nijman IJ, Beeke M, Hanekamp E, Lenstra JA (2004). Maternal and paternal lineages in cross-breeding bovine species. Has wisent a hybrid origin?. Mol Biol Evol.

[4] Gautier M, Moazami-Goudarzi K, Levéziel H, Parinello H, Grohs C, Rialle S (2016). Deciphering the wisent demographic and adaptive histories from individual whole-genome sequences. Mol Biol Evol.

[5] Wecek K, Hartmann S, Paijmans JLA, Taro U, Xenikoudakis G, Cahill JA, et al. Complex admixture preceded and followed the extinction of wisent in the wild. BioArxiv. 2016. http://biorxiv.org/content/early/2016/07/15/059527. 10.1093/molbev/msw254 PMC5356474 28007976

[6] Marris E. Mysterious origin of European bison revealed using DNA and cave art. http://www.nature.com/news/mysterious-origin-of-european-bison-revealed-using-dna-and-cave-art-1.20822. Accessed 8 Nov 2016.

[7] Shapiro B, Drummond AJ, Rambaut A, Wilson MC, Matheus PE, Sher AV (2004). Rise and fall of the Beringian steppe bison. Science.

[8] Lenstra JA, Felius M, Garrick D, Ruvinsky A (2015). Genetic aspects of domestication. The genetics of cattle.

[9] Qiu Q, Zhang G, Ma T, Qian W, Wang J, Ye Z (2012). The yak genome and adaptation to life at high altitude. Nat Genet.

[10] Qiu Q, Wang L, Wang K, Yang Y, Ma T, Wang Z (2015). Yak whole-genome resequencing reveals domestication signatures and prehistoric population expansions. Nat Commun.

